# The Effect of Virtual Reality Games on the Gross Motor Skills of Children with Cerebral Palsy: A Meta-Analysis of Randomized Controlled Trials

**DOI:** 10.3390/ijerph16203885

**Published:** 2019-10-14

**Authors:** Zhanbing Ren, Jinlong Wu

**Affiliations:** Department of Physical Education, Shenzhen University, Shenzhen 518061, China; 1800371011@email.szu.edu.cn

**Keywords:** virtual reality games, gross motor skill, cerebral palsy

## Abstract

This review aimed to systematically evaluate the rehabilitatitive effect of Virtual Reality Games (VRGs) for gross motor skills of children with cerebral palsy (CP), and to give scientific grounds for the formulation of rehabilitation therapy for these children. To this end, the literature in Chinese databases (CNKI and Wanfang Data) as well as the databases of other countries (Web of Science, PubMed, EBSCOhost, Informit, Scopus, Science Direct and ProQuest) from the establishment dates of these databases to June 3rd 2019 was retrieved in order to collect randomized controlled trials with regard to the intervention effect of VRGs and traditional therapy on gross motor skills of children with CP, and the literature was screened as per inclusion and exclusion criteria. The PEDro scale was then used to evaluate the methodological quality of the included literature, and the software Review Manager 5.3 was employed to analyze the combined effect size. As a result, 7 randomized controlled trials and 234 children with CP were included. Meta-analysis showed that VRGs could improve gross motor skills of children with CP. Combined effect size of gross motor skills *SMD* = 0.37 [95% CI = (0.06, 0.68), *p* = 0.02)]. In conclusion, the VRG intervention program can enhance gross motor skills of children with CP to some extent. In view of the limitations regarding methodologies and the quality and quantity of the literature in this research, more quality randomized controlled trials are needed so as to draw convincing conclusions of effect of VRG intervention on gross motor skill development of children with CP in future studies.

## 1. Introduction

Cerebral palsy (CP), a syndrome caused by non-progressive brain damage and developmental defects during the period from conception to infancy, mainly results in dyskinesia and postural abnormalities [1]. According to the statistics of World Health Organization, the incidence of CP in developed countries ranges from 0.2% to 0.3%. It is also reported that the prevalence of CP in 0- to 6-year-olds in China falls between 0.19% and 0.40% and reaches 0.56% in remote northwestern areas; the number of child cases in the whole country is approximately 6 million and increases by 45,000 people per year, which has become a major problem in public health [2]. The damage to the advanced central nervous system of children with CP may cause secondary injuries, such as physical spasm, amyotrophy, skeletal deformity, myasthenia and developmental coordination disorder, which constrain the children’s ability to move, thereby impacting upon their development of gross motor skills [3,4]. Research has shown that the failure to timely identify and fill the gap in the development of children’s gross motor skills may lead to deficiencies in motor ability and in other respects [5]. In addition, studies have proved that the gross motor skill disorder is an important factor hindering children with CP from participating in physical activities [6]. If not taking part in physical activities for a long time, these children will face higher risk of secondary psychological problems like pain, depression, social phobia and fatigue [7],and their physical fitness associated with muscle, such as strength, endurance and cardiopulmonary function, will often be poorer than that of normal children of the same age [8]. In this regard, it is all-important to pay attention to the development of the gross motor skills of children with CP.

Intensive motor training paradigms are often provided to develop the gross motor function of children with CP. Both quantity and quality of motor experience is important to the brain plasticity and functional recovery [9],so, to develop effective rehabilitation protocols to promote gross motor function recovery, timing and dose need to be considered. Some studies have indictaed that traditional centre-based CP rehabilitation (e.g., hospital, gymnasium, sports centre) programmes have shown positive effects for children with CP, 30–45 min sessions every day, which seems to be necessary for neuroplasticity [10,11,12,13]. The traditional centre-based method used in physiotherapy, such as group therapy and adjuvant therapy with a therapist, targets at a child with a certain type of CP on a face-to-face basis can intensify communication between children with CP and their parents [14]. However, it was often beyond the reach of the centre-based health care systems to offer more than 1–2 30–60 min sessions of physiotherapy (e.g.,active play with a coach, group activities with a therapist) pr week to children with CP due to time-comsuming and costly [15]. Therefore, it is necessary to look for cost-effective physiotherapy which can help children with CP to undergo intensive training for long time enough. Recently it has been found that home-based interactive training have positive rehabilitative effects for children with CP in improving balance performance [16],basic motor abilities, functional muscle strength and walking efficiency [12]. So, home-based task-oriented exercises are a useful addition to centre-based occupational therapy and physiotherapy to ensure more intensive and longer lasting exercises for children with CP [4].

Virtual Reality Games (VRGs) is a kind of whole-body interactive video game with the features of where people can physically immerse in a non-phsyical world through three-dimensional display at home [17].Such immersive experience in a safe, enjoyable, and playful environement is associated with less fatigue and more relaxation, which may attract children, including those with CP [18]. When children play games, actions involved, such as smiling, laughing, dancing and screaming, can intensify bioelectrical signals, including its circuits, in the brain [19]. Additionally, home-based whole-body interaction enables a heightened somatosensory experience in these games, where factors such as duration, intensity and repetition of the childrens’ activities may lead to improvement in their condition, gross motor function of children with CP can be promoted though home-based as well as centre-based CP rehabilitation [20,21,22]. VRGs as a interactive exercises auxiliary devices can keep children interested and taking repeated rehabilitation exercises; in contrast, the traditional centre-based treatment of dyskinesia needs the help of various games and facilities, such as bowling balls, obstacles, baskets of different heights, and takes large space. Apart from that, traditional centre-based rehabilitation therapy also needs (a) seasoned therapist(s) to communicate with and encourage children, so as to ensure smooth treatment by raising children’s excitement and interest. Compared with traditional scheme, VRG intervention can be performed in a smaller room, and the interaction of VRG device provides a challenging, encouraging and safe environment, so children with CP who are often unwilling to receive traditional therapy tend to choose VRG treatment [18,23], which will help with the motor skill development of children with CP. Due to practical and financial reasons home-based VRGs is becoming more important for rehabilitation of children with CP.

A literature review concerning children with CP has shown that VRG intervention can improve the gross motor skill development of these children, including their strength, balance, coordination and other physical qualities [24]. Nevertheless, the evidence of VRGs as a therapeutic intervention of CP is insufficient, with no quantitative studies indicating the effectiveness of VRGs in intervening in the development of the gross motor skills of children with CP. It has been found through the meta-analysis of the effect of VRGs on upper limb motor skills that VRGs in VR environment are feasible tools to improve the upper limb motor skills of children with CP [25]. Despite the improvement of gross motor skills of children with CP is indicated in the studies, experiments including the ones other than randomized controlled trials have shown limited appraisal evidence of it. A recent study has shown that VRGs have moderate positive effect on the gross motor skill development of children with developmental disorder [26],but no research by far has shown the effectiveness of VRGs on the gross motor skill development of children with CP. In this regard, this research aimed to explore the effect of VRGs on the gross motor skill development of children with CP by employing meta-analysis. In addition, the effects of VRGs intervention plan (including single intervention time, intervention frequency, intervention cycle, total intervention time) on the gross motor skill in children with CP are further determined. Therefore, it lays important theoretical groundwork for the effect of VRG-based treatment to gross motor skill development of children with CP, and provides a crucial decision-making basis for clinical staff.

## 2. Methods

### 2.1. Literature Retrieval

The review was conducted in accordance with the preferred reporting items for systematic review and meta-analysis (PRISMA) statement [27]. The literature in Chinese and English databases (CNKI, Wanfang Data, Web of Science, PubMed, EBSCOhost, Informit, Scopus, Science Direct and ProQuest) as of June 3^rd^ 2019 was retrieved, with “体感游戏” (active video gaming), “虚拟现实游戏” (VR game), “脑瘫儿童” (children with CP) and “脑性瘫痪儿童” (children with cerebral palsy) as Chinese keywords, and “exergame”, “virtual reality games”, “active video game”, “active video gaming”, “Wii”, “Play Station”, “Kinect”, “virtual reality”, “cerebral palsy”, “cerebral palsies”, “Little disease”, “infantile palsies”, “spastic diplegia(s)”, “spastic diplegic”, “spastic hemiplegia(s)”, “spastic hemiplegic”, “spastic quadriplegia(s)”, and “spastic quadriplegic” as English keywords.

### 2.2. Literature Inclusion and Exclusion Criteria

#### Inclusion Criteria and Exclusion Criteria

All randomized controlled trials in the literature should compare the influence of VRGs and traditional therapy over the gross motor skills of children with CP, evaluate its methodological quality, and integrate the experimental data. The following inclusion criteria were used to select articles for the meta-analysis: (1) The literature should be published, excluding conference abstracts. (2) The subjects of studies are children with CP(e.g., children with hemiplegic cerebral palsy, children with diplegic cerebral palsy, children with quadriplegia cerebral palsy). (3) VRGs are the main therapeutic interventions (e.g., VRGs and untrained, VRGs combined with traditional rehabilitation therapy and traditional rehabilitation therapy only). (4) The research should provide sample size, mean, standard deviation. (5) The outcome indicators are gross motor skills.

The following exclusion criteria were used to select articles for the meta-analysis:(1) In terms of titles and abstracts, theoretical research, systematic reviews, or the papers in which subjects were adults or the elderly, in which there was no control group in experiments, or in which other interventions were used, should be excluded. (2) From the perspective of the full text, the papers would also be excluded if it was non-RCT research, if subjects were over 18 years’ old or did not suffer from CP, if the outcome indicators failed to meet the requirements of analysis, or if result data were missing.

### 2.3. Literature Quality Evaluation

Kappa index is often used to analyze the consistency of two people’s (or two test methods’) opinions on the same subject. The general standard of judgment is: Kappa index > 0.75, better consistency; 0.75 ≤ Kappa index ≤ 0.4, good consistency; Kappa index < 0.4, poor consistency.

The risk of bias of the included studies was carried out by two independent assessors as per the PEDro scale [28]. In case of divergence of opinion between the two assessors, a third assessor would intervene. When reaching a consensus, the assessors would make a final decision on the quality of included studies. The following 11 criteria were taken into consideration (one point for each criterion): a. eligibility criteria were specified; b. subjects were randomly allocated to groups; c. allocation was concealed; d. the groups were similar at baseline regarding the most important outcome indicators; e. there was blinding of all subjects; f. there was blinding of all therapists; g. there was blinding of all assessors; h. measures of at least one key outcome were obtained from more than 85% of the subjects initially allocated to groups; i. all subjects for whom outcome measures were available received the treatment or, where this was not the case, data for at least one key outcome was analyzed by “intention to treat”; j. the results of between-group statistical comparisons were reported for at least one key outcome; k. the study provided both point measures and measures of variability for at least one key outcome. The highest mark for each study was 11: low risk of bias (=7 or more); moderate risk of bias (=5–6); high risk of bias (=4 or less).

### 2.4. Data Extraction and Analysis

In the process of retrieval, two searchers extracted the relevant indicators from the included literature using the independent and double-blind method. After reading papers, the following data were extracted: names of the first authors, years of publication, nationality, sample sizes, subjects, GMFCS level, subjects characteristics (number, gender and age), the intervention plan of VRG group (intervention method; intervention frequency; overall time of intervention), intervention location, platforms and types of VRG, outcome indicators. For outcome indicators, each RCT baseline as well as the mean (M) and standard deviation (SD) after intervention were extracted. If the research information that meets the inclusion criteria is incomplete, we will email or call the author in order to obtain accurate and objective real information.

Meta-analysis was applied to synthesize the eligible individual research results. The software Review Manager 5.3 was used in the analysis of outcome indicators of included literature to calculate effect size (the standardized mean difference (SMD) reflects the intervention effect of VRGs on gross motor skills in children with CP, and the random effects model [29] with 95% confidence interval (CI) was adopted. On top of that, *I^2^* was used to perform the heterogeneity test: when *I^2^* ≤ 25%, it indicates low heterogeneity in the studies; when 25% < *I^2^* ≤ 75%, it indicates moderate heterogeneity in the studies; when 75% < I^2^ ≤ 100%, sensitivity analysis should be performed to determine the sources of heterogeneity by deleting one literature at a time. Moreover, publication bias should then be evaluated using the funnel plot and Egger’s test [30].

## 3. Results

### 3.1. Retrieval Results

**Figure 1:**
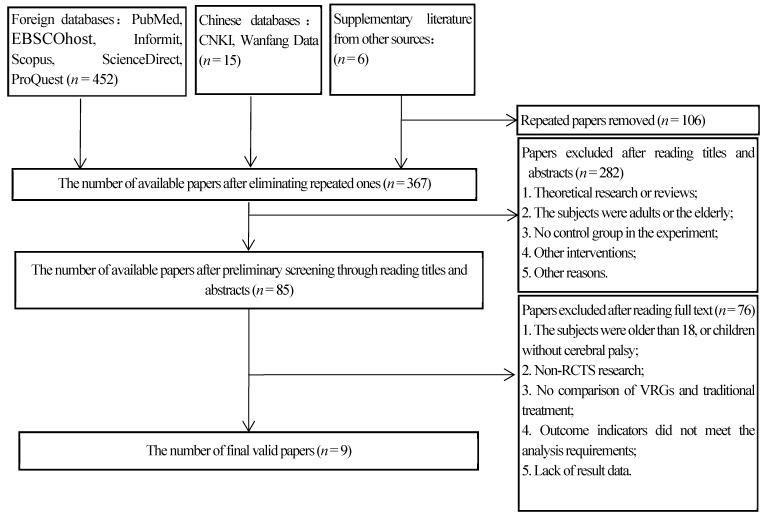
The flow chart of literature screening.

A total of 473 relevant papers were found through retrieval, including 65 from PubMed, 66 from EBSCOhost, 101 from Web of Science, 52 from InFormit, 34 from Scopus, 96 from Science Direct and 38 from ProQuest. As for Chinese databases, nine relevant papers were found in CNKI and six were found in Wan Fang Data. The overall number was reduced to 367 after repeated papers were deleted through NoteExpress 3.2.0 (AEGEAN, Beijing, China). In the light of their titles and abstracts, 85 papers were selected, and given the full text of these papers, 76 of them were excluded due to the failure to meet the inclusion criteria. The remaining 9 papers contain randomized controlled trials and the other contains randomized cross-over trial (Figure 1). The kappa index was 0.8734 for the abstract and 0.8932 for the full-papers.

### 3.2. Literature Characteristics

In this research, the nine included RTC papers were published between 2014 and 2019, including six English studies and three Chinese studies. Their characteristics are shown in Table 1. The subjects were children with CP, but there were some differences among these children (e.g., children with spastic diplegia and spastic hemiplegia; children with hemiplegic cerebral palsy; children with spastic diplegic cerebral palsy; children with spastic cerebral palsy; children with hemiplegia, diplegia and quadriplegia cerebral palsy; children with bilateral spastic cerebral palsy). One paper did not report the CP type of the children. In addition, the GMFCS levels of children with CP were also significantly different. Level 1 to 5 all can be found in the included papers. The numbers of samples in these papers all ranged from 18 to 98, with the total number being 404. Two papers did not mention participants’ genders [31,32], and one did not state intervention locations [33]. The intervention concerned a comparison between VRG therapy and control group (VRGs versus no intervention, VRGs combined with traditional rehabilitation therapy versus traditional rehabilitation therapy only, VRGs versus traditional rehabilitation therapy). Some differences existed in the intervention plan. Firstly, each intervention took 17 to 40 min. Secondly, the frequency of intervention varied from 2 to 7 times a week, and the total duration of intervention ranged from 400 to 2400 min. Finally, the intervention periods were also remarkably different, with the shortest being 4 weeks and some longer ones reaching 9 weeks.

There were also great differences among VRGs platforms. Besides the commonly used platform Nintendo, the Eloton SimCycle Virtual Cycling System, Mitii, Q4 situational interactive training system, the somatosensory games on Microsoft Xbox Kinect platform, TYROMOTION GmbH, and limb A2 feedback system were also included. Four papers did not state the VRG types that children involved [31,34,35,36]. Furthermore, seven of nine papers had one outcome indicator while three papers had two (D and E in the GMFM test) [32,33,34].

### 3.3. Quality Evaluation

For the quality assessment of included studies, the agreement among assessors was 92.5%. According to the PEDro scale, the methodological quality of all the experimental studies ranged from the score of 6 to 8. The higher the score, the lower the risk of bias (Table 2). 

Specifically speaking, seven studies were deemed to have low risk of bias and two studies were deemed to have moderate risk of bias. Since allocation concealment was not conducted in all the included studies, the quality of included studies decreased. On top of that, blinding was hard to carried out in the studies (it was only conducted in one study), so the score of blinding was deducted for most of the studies.

### 3.4. Result of Meta-Analysis

#### 3.4.1. Effects of VRG Group and Control Group on Gross Motor Skills of Children with CP

**Figure 2:**
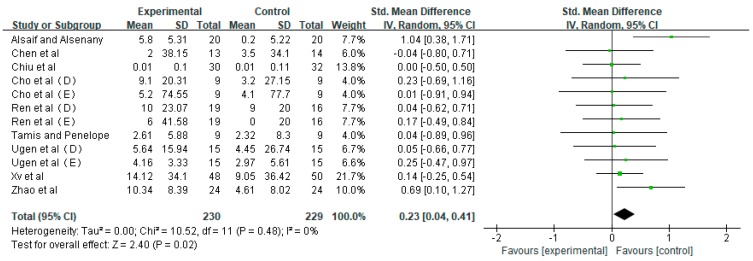
Effects of VRGs on Gross Motor Skills of Children with CP.

The nine papers reported the intervention effect of VRGs and traditional therapy on gross motor skills of children with CP, including 12 RCT data. The average effect size was 0.23 (random effect model), and the funnel plot showed no obvious asymmetry (Egger regression intercept = 0.344, *p* = 0.982). All RCT data were tested by meta-analysis, and the effects of VRG group and control group on gross motor skills in children with CP were evaluated with different measuring tools. The results showed that VRG intervention have remarkable effect on gross motor skills in children with CP (small effect, no heterogeneity, SMD = 0.23, *p* = 0.02, I2 = 0%) (Figure 2).

#### 3.4.2. Analysis of Moderating Effects

The results of subgroup analysis of the VRGs’ intervention plan on gross motor skills in children with CP were listed in Table 3. Single intervention time (*p* = 0.77), weekly intervention frequency (*p* = 0.15), intervention cycle (*p* = 0.64) and overall intervention time (*p* = 0.11) were found to have no significant impact.

## 4. Discussion

### 4.1. Meta-Analysis of the Intervention Effect of VRGs on Gross Motor Skills of Children with CP

There are many empirical studies on the intervention effect of VRGs on children’s physical health, mainly covering physical activities as well as psychological and motor development [40,41,42,43,44,45,46]. On top of that, VRGs are increasingly applied in the field of sports rehabilitation, and the intervention of VRGs in special children is centered on the mobility, balance and motor development of children with autism [46,47,48,49,50,51,52,53], CP [34,36,39,54,55]and developmental coordination disorder [18,26,56,57], without many studies focusing on relevant meta-analysis. This research mainly aims to comprehensively assess the effect of VRGs and traditional therapy on the gross motor skills of children with CP. The nine studies included were randomized controlled trials of VRGs and traditional therapy, and the subjects were all children with CP. The result of methodological quality evaluation suggested that the possibility of bias in the included studies was 0%, and the result of meta-analysis showed that the combined effect size *SMD* = 0.23 [95% CI = 0.04, 0.41, *p* = 0.02], indicating that compared with traditional therapy, VRG intervention could effectively improve the gross motor skills of children with CP. In addition, the results of subgroup analysis of the VRGs’ intervention plan on gross motor skills in children with CP suggest that single intervention time, weekly intervention frequency, intervention cycle and overall intervention time exert no significant impact. However, the combined effect size indicated that when the single intervention time was 17–40 min, the intervention frequency was more than 5 times per week, the intervention cycle was over 12 weeks and the overall intervention time was more than 1000 min, VRGs had a greater impact on the gross motor skills in children with CP. It is also noteworthy that there was heterogeneity among the included studies. Through the method of eliminating the studies one by one, after removing other individual studies, the combined effect also showed that VRG intervention could substantially improve the gross motor development of children with CP and showed no significant difference from the combined effect when all the studies were included, meaning that individual studies did not have major influence over results, although they did have certain impact upon outcome indicators.

### 4.2. Analysis of Influencing Factors of VRG Intervention on Gross Motor Skills of Children with CP

Gross motor activities, fulfilled by the torso, limbs and other big muscle groups, are basic motor skills developed in people’s childhood, an early stage of human life cycle, and they play a key role in children’s cognition and emotion development [58].In recent years, the effect of VRGs on children with CP has been verified through some experiments. This paper believes that VRGs could improve the motor balance of children with CP, which was mainly attributed to the fact that VRGs improved cerebral palsy children’s motor stability, postural control ability and the motor ability of the hemiplegic side.

#### 4.2.1. VRGs may Improve the Movement Stability of Children with CP

Gordand et al. [59] discussed the possibility of improving gross motor skills of children with CP through VRGs. Gradually becoming a rehabilitation method for children with CP, VRGs can not only keep children focused and moving repeatedly [23], but can also enhance their strength of motor muscles and improve their stability of movements. The integration of visual perception and motor skills is a perceptual integration ability developed in an early stage of childhood, which refers to the ability to coordinate visual perception with body movements [60]. The improvement of visual perception plays a positive role in the movement stability of children with CP. For example, Bilde et al. [4] found that the muscle strength and visual perception of children with CP were significantly improved after VRG intervention. Snider et al. [61] also believed that VRGs could better the visual perception and movement stability of children with CP. Cho et al. [32] performed treadmill VRG intervention on children with CP and found that the gross motor skills of children with CP were improved after intervention, and the improvement of muscle strength, vision, and motor skills is important for enhancing the movement stability of children with CP. To summarize, VRGs can improve the action control ability of children with CP, thereby enhancing their gross motor skills.

#### 4.2.2. VRGs may Improve the Postural Control Ability of Children with CP

Postural control ability is a key factor impacting upon the motor skills of children with CP [62]. A study found that children with CP, due to the lack of postural control ability, tended to fall down and face risks when performing certain actions [62]. Cho et al. [32] believed that VRGs improved the symmetry of the two sides of body for children with CP, so the center of gravity was evenly distributed in lower limbs, which increased the stability of standing, thereby improving postural control ability. In addition, another study also showed that VRG gait training intervention increased the strength of lower limbs of children with CP, thereby increasing the children’s postural control ability and finally improving their walking ability; the study also suggested that children with CP could transfer the skills they learned from VRGs to the real life [32]. Harris’ research indicated that VRGs as a rehabilitation method could not only provide a safe learning environment for children with CP, but could also help them master certain motor skills [23]. It can be seen that VRGs can improve the gross motor skills of children with CP by enhancing their postural control ability.

#### 4.2.3. VRGs may Improve the Motor Ability of Hemiplegic Side of Children with CP

In terms of the improvement of the hemiplegic side, studies like the one carried out by Chiu et al. [38] found that VRGs based on arm movements such as frisbee and bowling did not enhance gross motor skills of hands and arm strength of children with CP, but they noted through parents’ reports that these children’s arms on the hemiplegic side developed new movements (for instance, their forearms could rotate outward). Surprisingly, Urgent et al. [33] found that compared with the children undergoing traditional therapy, the children with CP in the VRG group managed to jump remarkably more times with the leg on the hemiplegic side, especially in ski jumping and racing games. From the perspective of treatment effect of experiments, VRG intervention allowed children with CP to effectively improve their limb function of the hemiplegic side, which could help them to develop gross motor skills. Moreover, Zhao et al. [39] found that VRG schemes based on movement observation training could substantially advance the motor performance of children with CP. Similarly, the research conducted by Buccinod et al. [63] showed that movement observation-based VRG schemes could enhance the motor skills of upper limbs of children with CP.

To sum up, more and more studies have applied VRG intervention to children with CP and obtained preliminary results from postural control and gait improvement to gross motor skill development of children with CP. This research has found that VRGs can play a positive role in the improvement of the gross motor skills of children with CP. With the development of various VRGs, new approaches to children’s CP treatment are presented in front of clinical staff. VRGs as an intervention approach still needs further research due to existing methodology and studies limitations. Additionally, the research focus in the future will also lie in the comparison of effect of various interventions on children with CP to reveal the difference among them.

## 5. Research Limitations

This research has some limitations. To start with, there are only nine research studies included in this overwiew, so the sample size is not large enough, and the included studies may not be well-rounded; some conference papers and unpublished papers were not included, so there may exist publication bias [64]. Besides, included studies contain multiple interventions and measurement results. On top of that, the included studies are limited by insufficient descriptions of randomized control and blinding, and some data regarding the measurement in various fields in some experiments may be lost [65]. Moreover, this paper only takes the children with CP as the subjects, and does not distinguish among specific types of CP and different GMFCS levels. The most important limitation of this study is that the included papers involves more than one treatment, so it may be difficult to make clear that all the improvements come from the intervention of VRGs. Some papers do not match the search terms, but are located in the field that might be missed in this study [66]. Based on the above reasons, the intervention effects of VRGs on the gross motor skills of children with CP may be overrated. In the future, more rigorous and reliable randomized controlled trials are needed to accurately determine the long-term effects of VRGs on gross motor skills of children with CP.

## 6. Conclusions

Preliminary evidence shows that VRGs have positive effect on the improvement of gross motor skills of children with CP. Additionally, the single intervention time was 17–40 min, the intervention frequency should be over 5 times per week, the intervention cycle was over 12 weeks while the total intervention time should be more than 1000 min. VRGs essentially belongs to the functional training that based on the ecological theory [67] and dynamical systems theory [68], which focus on the role of the environment and the task in the performance of functional activities.The merits of functional training have been affirmed by the therapists for its advantage of more purposeful, more functional and more enjoyable for the children [14].In this case, VRGs as one of the home-based functional training auxiliary devices would have a greater impact on gross motor skills in children with CP. However, due to the limitation of current methodologies and the number of existing studies, the research results should be taken with caution. As for the VRGs is concerned, how to deliver and supervise children with CP rehabilitation between centre-based system and home-based system still need further research.

## Figures and Tables

**Table 1:** List of basic characteristics of the included documents.

Researchers	Country: Language	Subjects	GMFCS Level (Number)		Characteristics of Subjects (Number; Genders; Ages)	Intervention Methods; Frequency of the Intervention Group; Total Time of the Intervention Group	Intervention Location	VRGs Platform	VRGs Category	Outcome Indicators
			Experimental Group	Control Group						
Chen et al. [37]. (2012)	Taiwan; English	Spastic diplegia and spastic hemiplegia children with CP	Level 1: 10; Level 2: 3	Level 1: 11; Level 2: 3	27; experimental group: 13; 9 male (8.7 ± 2.2) ; control group: 14; 9 (8.6 ± 2.2)	Warm-up exercise + sitting training + VRGs treatment; 20 min each time, 3 times a week for 12 weeks; 720 min	Rehabilitation Center	The Eloton SimCycle Virtual Cycling System (Eloton, Inc., NV, USA),	Virtual bicycle	(1)
Chiu et al. [38]. (2014)	Taiwan; English	Hemiplegic children with CP	level 1–3: 21 level 4–5: 11	level 1–3: 21 level 1–3: 9	62; experimental group: 32;15 male; (9.4 ± 1.9) Control group: 30; 13 male; (9.5 ± 1.9)	Routine rehabilitation + VRGs; 40 min, 3 times a week for 6 weeks; 720 min	Family	Nintendo wii	Bowling; flying disc; Aerial movement	(2)
Alsaif and Alsenany [31]. (2015)	Saudi-Arabia; English	Spastic diplegia children with CP	Level 3: 20	Level 3: 20	40; experimental group: 20; Control group: 20; Age: 6–10 (NSL)	VRGs; 20 min a day, 7 times a week for 12 weeks; 1680 min	Family	Nintendo wii-fit balance board game	Unspecified specific type	(3)
Ren et al. [34]. (2016)	China; Chinese	Spastic diplegia children with CP	Level 1: 8; Level 2: 11	Level 1: 7; Level 2: 9	35; experimental group: 19; 11 male; (53.88 ± 13.58) ; control group: 16; 9 (56.53 ± 9.67)	Routine rehabilitation + VRGs; 40 min each time, 5 times a week for 12 weeks; 2400 min	Hospital	Q4 Situational Interactive Training System (produced by Aomai, model q4)	Unspecified specific type	(4) (5)
Rgen et al. [33]. (2016)	Turkey; English	Spastic hemiplegia children with CP	Level 2: 15	Level 2; 15	30; experimental group: 15; 7 male; (11.07 ± 2.37); Control group: 15; 7 male; (11.33 ± 2.19)	Routine rehabilitation + VRGs ; 40 min each time, 2 times a week for 9 weeks; 720 min	Not clear	Nintendo wii-fit	Jogging plus, penguin slide, heading, ski jump, snowball fight, tilt city, perfect 10, and segway circuit play.	(4) (5)
Cho et al. [32]. (2016)	South Korea; English	Sputum children with CP	Level 1: 3; Level 2: 1; Level 3: 5	Level 1: 3; Level 2: 2; Level 3: 4	18; experimental group: 9, (10.2 ± 3.); Control group: 9, (9.4 ± 3.8) (NSL)	VRGs; 30 min a day, 3 times a week for 8 weeks; 720 min	Outpatient department	Nintendo wii-fit	Virtual reality treadmill	(4) (5)
Zhao et al. [39]. (2018)	China; Chinese	Spastic quadriplegia, spastic diplegia and spastic hemiplegia children with CP	Level 1: 18; Level 2: 6	Level 1: 17; Level 2: 7	48; experimental group: 24 ;11 male; (59.38 ± 11.29) months Control group: 24 people (male 16 female 8); (54.33 ± 10.93) months	Routine rehabilitation + VRGs; 40 min each time, 5 times a week for 5 weeks; 1000 min	Hospital	Microsoft Xbox kinect virtual reality platform	Dance music imitation	(6)
Pin and Butler [36] (2019)	China (Hong Kong); English	Spastic diplegia children with CP	Level 3: 8; Level 4: 1	Level 3: 8; Level 4: 1	18; experimental group: 9 people 5 male; (8.92 ± 2.25) Control group: 9 people (male 6 female 3); (9.59 ± 1.87)	Routine rehabilitation + VRGs; 20 min each time, 4 times a week for 6 weeks; 480 min	School	(TYROMOTION GmbH, Graz, Austria)	Unspecified specific type	(1)
Xv et al. [35]. (2019)	China; Chinese	Children with CP	Not clear	Not clear	98; experimental group: 50; 22 male; (45.01 ± 8.25) months Control group: 48; 22 male; (43.87 ± 8.25) months	tDCS + VRGs; 20 min each time, 5 times a week for 4 weeks; 400 min	Hospital	Limb a2 feedback system (Guangzhou)	Unspecified specific type	(6)

Note: NSL: The male to female ratio is not mentioned; GMFCS: Gross Motor Function Classification System; VRGs: virtual reality games; (1) Gross Motor Function Measure-66; (2) hand dex with 9-hole peg test; (3) Movement Assessment Battery for Children (Hand flexibility, aiming and grasping, balance); (4) Gross Motor Function Measure-D; (5) Gross Motor Function Measure-E; (6) Gross Motor Function Measu.

**Table 2:** Methodological Quality Assessment for Included Studies.

Included Studies	A	B	C	D	E	F	G	H	I	J	K	Score
Chen et al. [37] (2012)	Yes	Yes	No	Yes	No	No	No	Yes	No	Yes	No	6/11
Chiu et al. [38] (2014)	Yes	Yes	No	Yes	Yes	Yes	No	Yes	Yes	Yes	No	8/11
Alsaif et al. [31] (2015)	Yes	Yes	No	Yes	No	No	No	Yes	Yes	Yes	No	6/11
Ren et al. [34] (2016)	Yes	Yes	No	Yes	No	No	No	Yes	Yes	Yes	No	6/11
Rgen et al. [33] (2016)	Yes	Yes	No	Yes	No	No	No	Yes	Yes	Yes	No	5/11
Cho et al. [32] (2016)	Yes	Yes	No	Yes	No	No	No	Yes	Yes	Yes	No	6/11
Zhao et al. [39] (2018)	Yes	Yes	No	Yes	No	No	No	Yes	Yes	Yes	No	6/11
Pin et al. [36] (2019)	Yes	Yes	No	Yes	No	No	No	Yes	Yes	Yes	No	7/11
Xv et al. [35] (2019)	Yes	Yes	No	Yes	No	No	No	Yes	Unclear	Yes	No	5/11

Note: a. eligibility criteria were specified; b. subjects were randomly allocated to groups; c. allocation was concealed; d. the groups were similar at baseline regarding the most important outcome indicators; e. there was blinding of all subjects; f. there was blinding of all therapists; g. there was blinding of all assessors; h. measures of at least one key outcome were obtained from more than 85% of the subjects initially allocated to groups; i. all subjects for whom outcome measures were available received the treatment or, where this was not the case, data for at least one key outcome was analyzed by “intention to treat”; j. the results of between-group statistical comparisons were reported for at least one key outcome; k. the study provided both point measures and measures of variability for at least one key outcome.

**Table 3:** Moderating Effects Analysis of VRGs Group’s Intervention Plan in Children with CP.

Intervention Plan	Level	Data	SMD	95%CI	I^2^	Intergroup Difference
						*p* Value
Single Intervention Time	≥17 min; <40 min	6	0.26	−0.07, 0.60	0%	0.77
	≥40 min	6	0.20	−0.05, 0.46	0%	
Intervention Frequency	≥2 times per week; <5 times per week	7	0.06	−0.21, 0.34	0%	0.15
	≥5 times per week	5	0.40	0.04, 0.75	0%	
Intervention Cycle	≥4 weeks; <12 weeks	8	0.19	−0.03, 0.41	0%	0.64
	≥12 weeks	4	0.32	−0.18, 0.81	0%	
Overall Intervention Time	≥400 min; <1000 min	8	0.09	−0.14, 0.32	0%	0.11
	≥1000 min	4	0.49	0.05, 0.94	0%	
